# Supplementary material for: Laniakea: an open solution to provide Galaxy “on-demand” instances over heterogeneous cloud infrastructures
Source: Gigascience. 2020 Apr 6;9(4):giaa033. doi: 10.1093/gigascience/giaa033 (PMC7136032; doi:10.1093/gigascience/giaa033)
Supplement: giaa033_GIGA-D-20-00009_Original_Submission [file giaa033_giga-d-20-00009_original_submission.pdf]

GigaScience

Laniakea: an open solution to provide Galaxy “on-demand” instances over heterogeneous cloud infrastructures.

--Manuscript Draft--

Manuscript Number:	GIGA-D-20-00009
Full Title:	Laniakea: an open solution to provide Galaxy “on-demand” instances over heterogeneous cloud infrastructures.
Article Type:	Research
Funding Information:	H2020 European Research Council (RIA 653549) — Not applicable
Abstract:	Background<br>While the popular workflow manager Galaxy is currently made available through several publicly accessible servers, there are scenarios where users can be better served by full administrative control over a private Galaxy instance, including, but not limited to, concerns about data privacy, customisation needs, prioritisation of particular job types, tools development, training activities. In such cases, a cloud-based Galaxy virtual instance represents an alternative that equips the user with complete control over the Galaxy instance itself without the burden of the hardware and software infrastructure involved in running and maintaining a Galaxy server.<br>Results<br>We present Laniakea, a complete software solution to set up a “Galaxy on-demand” Platform as a Service (PaaS). Building on the INDIGO-DataCloud software stack, Laniakea can be deployed over common cloud architectures usually supported both by public and private e-infrastructures. The user interacts with a Laniakea-based service through a simple front-end that allows a general setup of a Galaxy instance, then Laniakea takes care of the automatic deployment of the virtual hardware and the software components. At the end of the process, the user gains access with full administrative privileges to a private, production-grade, fully customisable, Galaxy virtual instance, and to the underlying virtual machine (VM). Laniakea features deployment of single-server or cluster-backed Galaxy instances, sharing of reference data across multiple instances, data volumes encryption, and support for VM image-based, Docker-based, and Ansible recipes-based Galaxy deployments. A Laniakea based Galaxy on-demand service, named Laniakea@ReCaS, is currently hosted at the ELIXIR-IT ReCaS cloud facility.<br>Conclusions<br>Laniakea offers to scientific e-infrastructures a complete and easy-to-use software solution to provide a Galaxy on-demand service to their users. Laniakea-based cloud services will help in making Galaxy more accessible to a broader user base by removing most of the burdens involved in deploying and running a Galaxy service. In turn, this will facilitate the adoption of Galaxy in scenarios where classic public instances do not represent an optimal solution. Finally, the implementation of Laniakea can be easily adapted and expanded to support different services and platforms beyond Galaxy.
Corresponding Author:	Federico Zambelli<br><br>ITALY
Corresponding Author Secondary Information:	
Corresponding Author's Institution:	
Corresponding Author's Secondary Institution:	
First Author:	Marco Antonio Tangaro
First Author Secondary Information:	
Order of Authors:	Marco Antonio Tangaro

Powered by Editorial Manager® and ProduXion Manager® from Aries Systems Corporation

	Giacinto Donvito
	Marica Antonacci
	Matteo Chiara
	Pietro Mandreoli
	Graziano Pesole
	Federico Zambelli
**Order of Authors Secondary Information:**	
**Additional Information:**	
**Question**	**Response**
Are you submitting this manuscript to a special series or article collection?	No
**Experimental design and statistics**<br><br>Full details of the experimental design and statistical methods used should be given in the Methods section, as detailed in our Minimum Standards Reporting Checklist. Information essential to interpreting the data presented should be made available in the figure legends.<br><br>Have you included all the information requested in your manuscript?	Yes
**Resources**<br><br>A description of all resources used, including antibodies, cell lines, animals and software tools, with enough information to allow them to be uniquely identified, should be included in the Methods section. Authors are strongly encouraged to cite Research Resource Identifiers (RRIDs) for antibodies, model organisms and tools, where possible.<br><br>Have you included the information requested as detailed in our Minimum Standards Reporting Checklist?	Yes
**Availability of data and materials**<br><br>All datasets and code on which the	Yes

conclusions of the paper rely must be either included in your submission or deposited in publicly available repositories (where available and ethically appropriate), referencing such data using a unique identifier in the references and in the "Availability of Data and Materials" section of your manuscript.<br><br>Have you have met the above requirement as detailed in our Minimum Standards Reporting Checklist?	

Manuscript
Click here to download Manuscript Laniakea_GigaScience_RELOADED_final.docx

Laniakea: an open solution to provide Galaxy "on-demand" instances over heterogeneous cloud infrastructures.

Marco Antonio Tangaro[1], Giacinto Donvito[2], Marica Antonacci[2], Matteo Chiara[3], Pietro Mandreoli[3], Graziano Pesole[1,4], Federico Zambelli[1,3]

1. Institute of Biomembranes, Bioenergetics and Molecular Biotechnologies, National Research Council (CNR), Via Amendola 165/A, 70126 Bari, Italy
2. National Institute for Nuclear Physics (INFN), Section of Bari, Via Orabona 4, 70126 Bari, Italy
3. Department of Biosciences, University of Milan, via Celoria 26, 20133 Milano, Italy
4. Department of Biosciences, Biotechnologies and Biopharmaceutics, University of Bari, Via Orabona 4, 70126 Bari, Italy

Abstract

Background
While the popular workflow manager Galaxy is currently made available through several publicly accessible servers, there are scenarios where users can be better served by full administrative control over a private Galaxy instance, including, but not limited to, concerns about data privacy, customisation needs, prioritisation of particular job types, tools development, training activities. In such cases, a cloud-based Galaxy virtual instance represents an alternative that equips the user with complete control over the Galaxy instance itself without the burden of the hardware and software infrastructure involved in running and maintaining a Galaxy server.

Results
We present Laniakea, a complete software solution to set up a "Galaxy on-demand" Platform as a Service (PaaS). Building on the INDIGO-DataCloud software stack, Laniakea can be deployed over common cloud architectures usually supported both by public and private e-infrastructures. The user interacts with a Laniakea-based service through a simple front-end that allows a general setup of a Galaxy instance, then Laniakea takes care of the automatic deployment of the virtual hardware and the software components. At the end of the process, the user gains access with full administrative privileges to a private, production-grade, fully customisable, Galaxy virtual instance, and to the underlying virtual machine (VM). Laniakea features deployment of single-server or cluster-backed Galaxy instances, sharing of reference data across multiple instances, data volumes encryption, and support for VM image-based, Docker-based, and Ansible recipes-based Galaxy deployments. A Laniakea based Galaxy on-demand service, named Laniakea@ReCaS, is currently hosted at the ELIXIR-IT ReCaS cloud facility.

Conclusions
Laniakea offers to scientific e-infrastructures a complete and easy-to-use software solution to provide a Galaxy on-demand service to their users. Laniakea-based cloud services will help in making Galaxy more accessible to a broader user base by removing most of the burdens involved in deploying and running a Galaxy service. In turn, this will facilitate the adoption of Galaxy in scenarios where classic public instances do not represent an optimal solution. Finally, the

implementation of Laniakea can be easily adapted and expanded to support different services and platforms beyond Galaxy.

Background

The recent improvements in our capacity to gather vast amounts of complex, multi-layered and interconnected biomolecular data demand a parallel development and enhancement of the computational tools that are employed to analyse and handle this wealth of information. On the other hand, the rapid proliferation of those tools can make the execution of complex bioinformatics workflows cumbersome due to, among other things, a profusion of data formats, long and convoluted command lines, versioning, and the need to handle and store multiple intermediate files. In turn, frequently, this makes harnessing the information contained in the biomolecular data onerous even for expert bioinformaticians, which currently represent a limited resource [1–3]. Furthermore, the complexity of bioinformatics analyses often poses a significant obstacle to reproducibility [4] as well as being an intimidating barrier for biologists aspiring to explore their data in autonomy [5], students [6], and healthcare operators planning to adopt *clinical bioinformatics* approaches within their medical protocols [7]. For these reasons, in the past few years, considerable effort has been put in the development of workflow manager software platforms for bioinformatics (see [8] for a review). Usually, these platforms do not only provide a more user-friendly work environment but also improve reproducibility, facilitate data sharing, and enable collaborative data processing.

Galaxy

The Galaxy platform is one of the most successful examples of such workflow management software. Indeed, by providing a consistent, user-friendly, flexible, practical, and customisable gateway to a vast array of bioinformatics software and analysis workflows, Galaxy has attracted a vast and thriving community of users [9]. The software consists of an open-source server-side application accessible through a simple web interface that serves as a gateway to a wealth of tools for datasets handling and analysis, workflow design, visualisation, and sharing of results. Although from the user's perspective using a Galaxy service to run bioinformatics analyses is pretty straightforward, the deployment of a production-grade Galaxy instance can require the configuration and maintenance of an extensive collection of helper components (e.g., database management system, web server, load balancer) and an even more extensive collection of bioinformatic tools and reference data. These issues coupled with the need for an adequate IT infrastructure required to properly support a Galaxy service have usually restricted the role of Galaxy service providers to institutions or groups with suitable IT facilities and appropriate technical know-how. At the time of writing, about 125 Public Galaxy instances are available (https://galaxyproject.org/use/), including the Galaxy Project flagships usegalaxy.org, usegalaxy.eu, and

usegalaxy.au, serving a vast community of users [9]. These are very welcome and useful resources. However, the public nature of the service implies some hardly addressable shortcomings like, for example, limited user quotas for computing and storage resources, lack of customisation options -e.g., installing or implementing new Galaxy tools or linking the instance to local data repositories-, and potential concerns for data security and privacy, a worry particularly noticeable when processing sensitive data. These considerations can, in turn, limit or outright interdict the use of Galaxy public instances for some specific applications, e.g., analyses requiring substantial computational resources, precision medicine [10,11], development or porting of new tools into Galaxy, training and teaching activities [12].

Cloud solutions to Galaxy provision

The cloud computing model [13] is rapidly gaining popularity within the life sciences [14–19] and the biomedical [7,20,21] communities. Among other advantages, cloud computing offers solutions and features that can overcome or mitigate the limitations of Galaxy public instances discussed in the previous section. Several efforts have already been put forward in this regard. Globus Genomics [22] provides a Galaxy-based bioinformatics workflow platform, built on Amazon cloud services, for large-scale next-generation sequencing analyses. CloudMan [23,24] allows the deployment of personal Galaxy instances relying on arbitrarily sized compute clusters on the Amazon cloud infrastructure (and can support OpenStack and OpenNebula through custom deployments). In 2017 CloudMan merged forces with the Genomic Virtual Laboratory (GVL) [25], providing a cloud-based suite of genomics analysis tools, including Galaxy, that can be deployed on OpenStack based clouds as well as on AWS and Azure. PhenoMeNal [26] is a recent effort to develop a Cloud Research Environment for metabolomics, including Galaxy among several other software. A further example is provided by Krieger and colleagues that describe a possible configuration stack to deploy Galaxy on an OpenStack based IaaS (Infrastructure as a Service) [27].

The attractiveness of Galaxy cloud solutions is made evident also in the 2016 Galaxy update [28] reporting that over 2,400 Galaxy servers were launched on the Amazon cloud in 2015 alone, pointing to a strong demand for ready-to-use but private virtual Galaxy instances. The Amazon Galaxy service [29] is, however, a commercial solution that can discourage researchers or healthcare facilities from adopting it due to funding or budget issues, ethical concerns, or legal requirements (e.g., EU General Data Protection Regulation). Finally, an interesting point that has emerged only recently is how complex scientific user requirements, as can be considered bioinformatics workflows, can be better served by federated cloud solutions that bind together distinct and heterogeneous cloud infrastructures rather than by single cloud providers (see for example [30,31] among others).

Overview

Hereby we introduce Laniakea (https://laniakea-elixir-it.github.io/), a software framework for the provision of *on-demand* Galaxy instances over federated cloud e-infrastructures. Laniakea can be instantiated on existing scientific e-infrastructures leveraging the open and modular architecture developed in the context of the INDIGO-DataCloud H2020 project [32,33] (INDIGO from now on). By hiding the technical complexity behind a user-friendly web front-end, Laniakea allows its users to configure and deploy virtual Galaxy instances with a handful of clicks, obtaining full administrative-level access both to the Galaxy instance and the underlying virtual hardware. Any Galaxy instance generated by Laniakea is accessible from a public IP address and retains all the functionalities and properties of a local Galaxy server. That is, the administrator (usually referred to as *admin user*) can install and configure new tools (e.g., through Galaxy Tool Sheds), add users -that are not required to be also users of the Laniakea service-, turn the instance public, configure jobs handling, manage jobs, establish user quotas and perform any other operation commonly available to Galaxy admin users.

Laniakea supports the deployment of Galaxy instances over three virtual hardware layouts: single node, static cluster, and elastic-cluster, that is cluster with workload-dependent automatic scaling of the number of nodes. Laniakea can deploy Galaxy instances starting from VM snapshots, we refer to it as Express for brevity, Docker containers [34], or using Ansible recipes (https://docs.ansible.com/ansible/latest/index.html). We refer to the latter case as “Live build”, since the entire Galaxy software stack is installed from scratch over a bare virtual machine: all the software components, including Galaxy tools, are retrieved on-the-fly from their respective online repositories. Live build introduces a flexible alternative compared to the somewhat more cumbersome VM snapshots and Docker containers. In fact, Ansible recipes make it possible to describe, and replicate, a complete software environment using just tiny text files, while VM snapshots and Docker containers usually weight several GBs.

Laniakea integrates for the first time, at the best of our knowledge on a Galaxy on-demand platform, a built-in technology to encrypt storage volumes at the filesystem level. Encrypted volumes allow the insulation of data from unauthorized access both from malicious attackers or trusted users of the same cloud infrastructure, notably including the administrators of the cloud and hardware layers. The user can activate and manage volume encryption in a straightforward manner, directly from the web front-end. Galaxy instances generated by Laniakea can also be automatically linked to local or remote CernVM-FS (CVMFS) repositories hosting reference data, meaning that all the

instances created by a Laniakea cloud service can be easily linked to the same read-only storage volume hosting reference data, either local or remote, in order to avoid unnecessary redundancy resulting in waste of storage resources.

Other functionalities of Laniakea stem from its INDIGO foundations and include support for a wide array of cloud managers, transparent orchestration of virtual hardware at different sites over a federated cloud infrastructure depending on where resources are available to the user and compatibility with the INDIGO-IAM [35] and ELIXIR-AAI [36] AAI services.

Fig. 1 provides an overview of the architecture of Laniakea. A step-by-step installation guide of the whole architecture is available at (https://laniakea.readthedocs.io/en/latest/index.html). Table S1 provides a brief summary of the principal Laniakea functions, aligning them to similar features of PhenoMeNal and GVL.

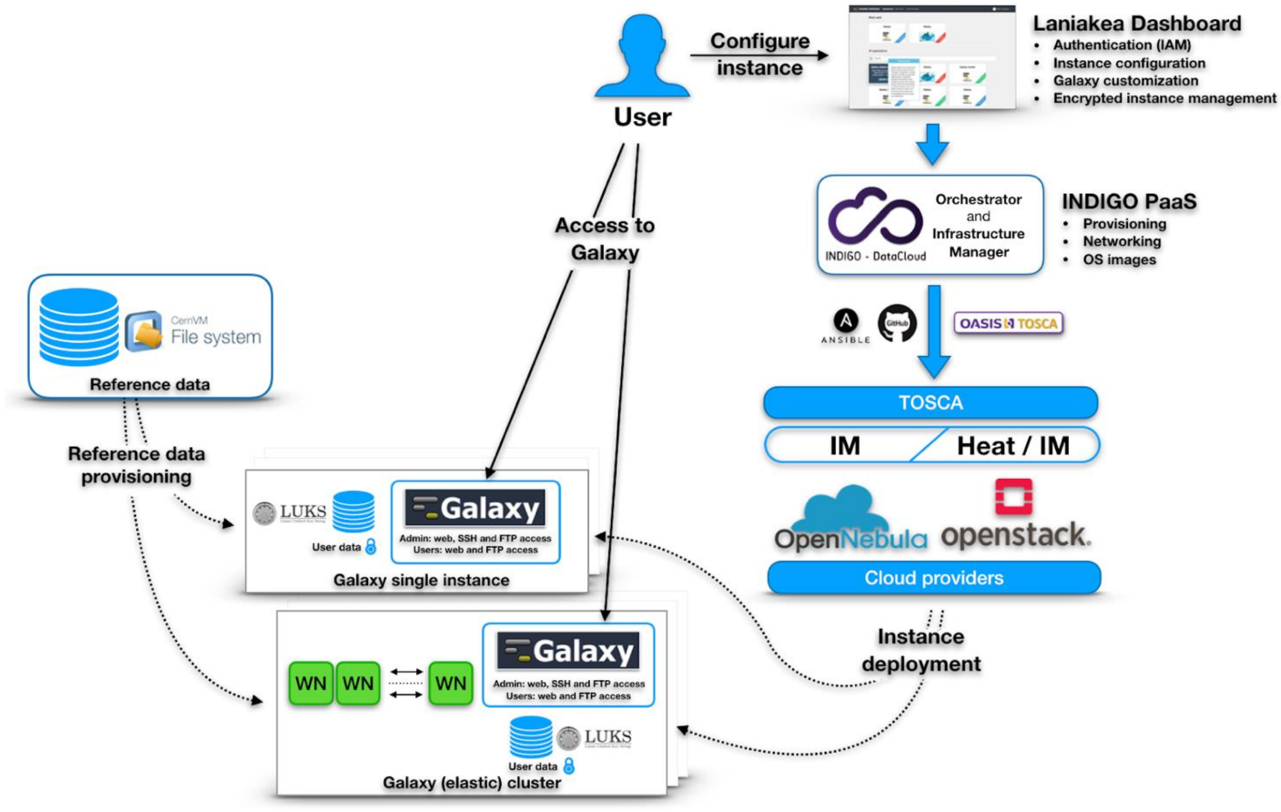

Fig. 1. Laniakea Architecture. The Laniakea Dashboard is the front-end that users access to configure and manage Galaxy instances. When a new Galaxy instance is requested by a user, the resulting TOSCA template is sent to the PaaS layer that employs INDIGO services to deploy the instance over the IaaS, retrieving the needed virtual hardware, storage, and networking resources. Finally, the Galaxy instance is configured with the requested set of tools (flavour), attached to a plain or LUKS encrypted storage volume, and to the CernVM-FS shared volume hosting reference data. At the end of the process, a public IP address is assigned to the freshly minted Galaxy instance is and made available to the user.

Laniakea Dashboard

The Laniakea Dashboard is the web front-end of Laniakea. The home page (Fig. 2) presents the selection of the available Galaxy virtual hardware layouts (i.e., single node, cluster, elastic cluster) and deployment strategies (i.e., Express, Live Build, or Docker).

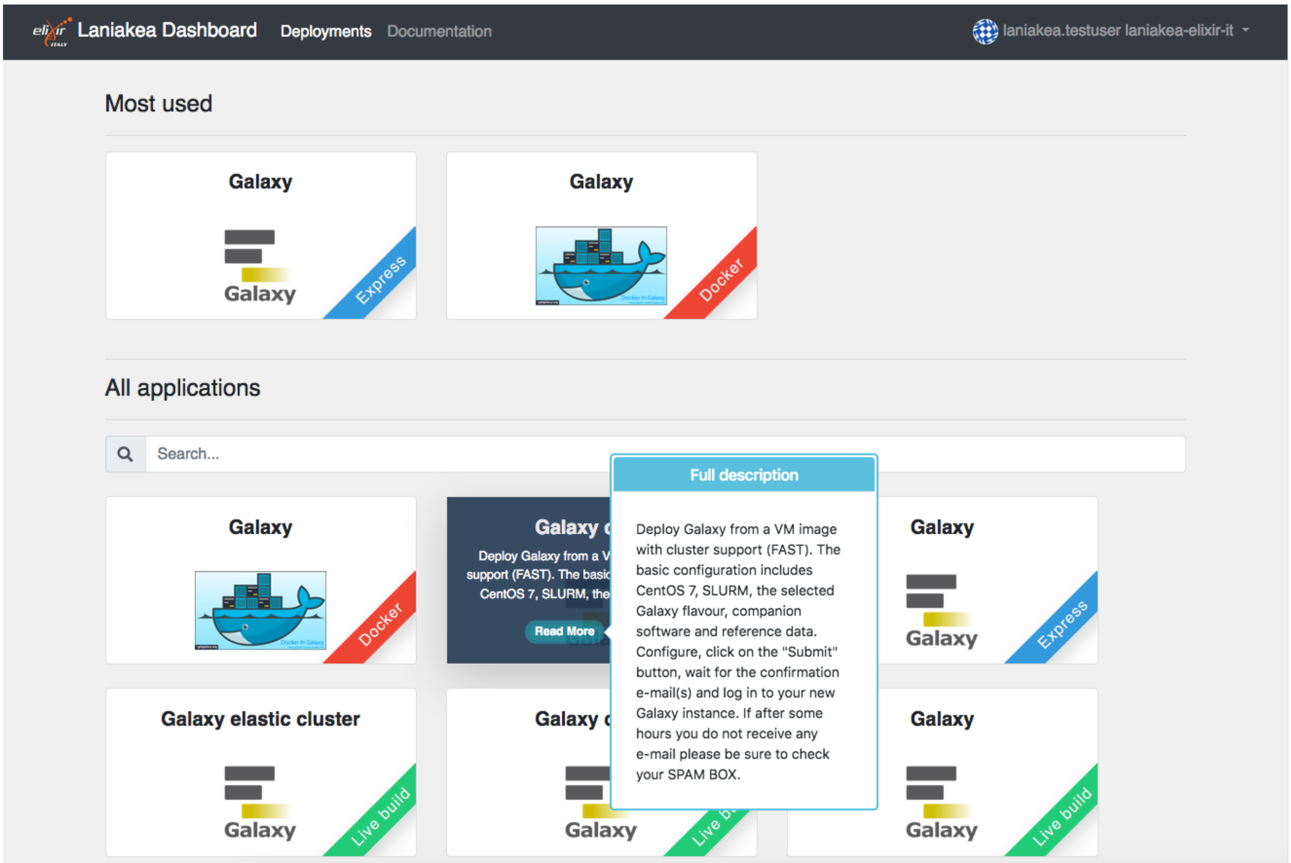

Fig. 2: Laniakea Dashboard home page. Each tile provides a quick explanation of the corresponding application and links to the configuration panels (see Fig. 3). Deployments using VM snapshots correspond to the tiles labeled as "Express". Deployments using Docker containers correspond to the tiles labeled as "Docker". Finally, deployments using Ansible recipes correspond to the tiles labeled as "Live build".

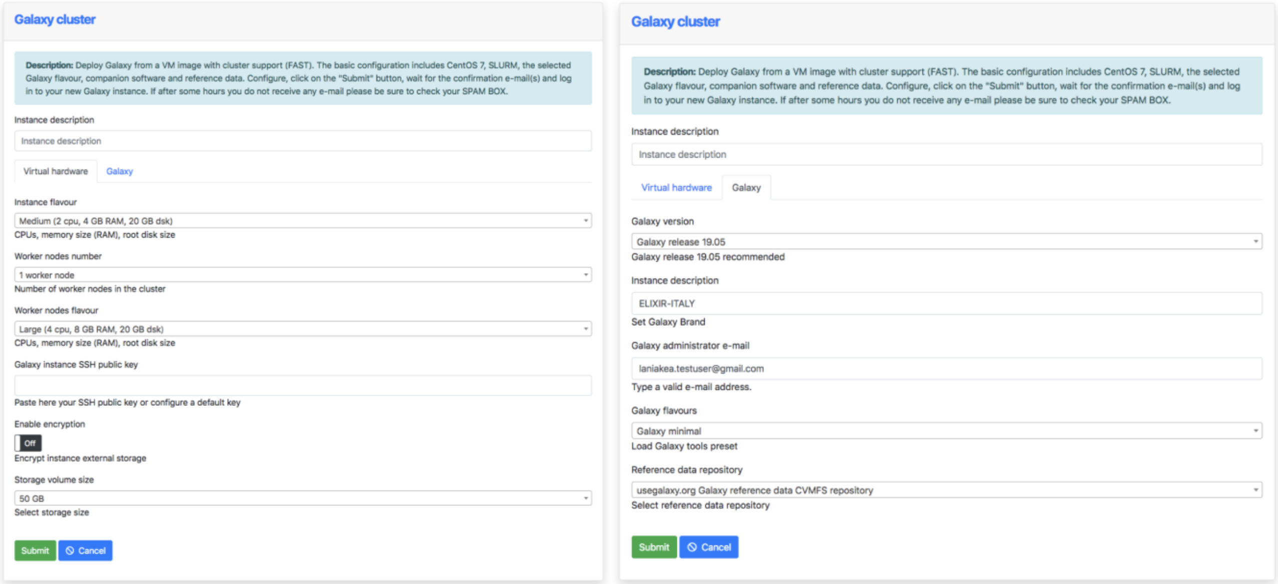

Fig 3: Laniakea Dashboard configuration panels. The "Virtual hardware" tab (left) allows the selection of the virtual hardware in terms of number of virtual CPUs, amount of RAM, size of the data volume (encrypted or not), number and hardware configuration of the worker nodes (only for cluster deployments), and requires the public SSH key of the user. The "Galaxy" tab (right) is used to tweak the software configuration: Galaxy version, description of the instance, the mail address of the administrator, Galaxy flavour (see Galaxy flavours), and reference data repository.

The configuration front-end is composed of two main panels (Fig. 3) that drive the user through the setup procedure of a new Galaxy instance:

- The Virtual Hardware configuration panel exposes the array of available hardware setups in terms of the number of virtual CPUs and amount of RAM. Users are asked to provide a valid SSH public key at this point, which can also be retrieved from the "SSH keys management" function of the Dashboard (Fig. S1). The SSH key is used to grant full access to the Virtual Machine hosting the Galaxy instance. The data storage volume size and type, i.e., plain or encrypted, are also selected at this stage. If the user selects a cluster backed Galaxy deployment, the panel allows for the setup both of the front-end and worker nodes.
- The Galaxy Configuration panel allows the selection of the Galaxy release version among the ones supported, the reference data CVMFS volume (when more than one is available), the e-mail of the instance administrator, and the Galaxy flavour (see Galaxy flavours).

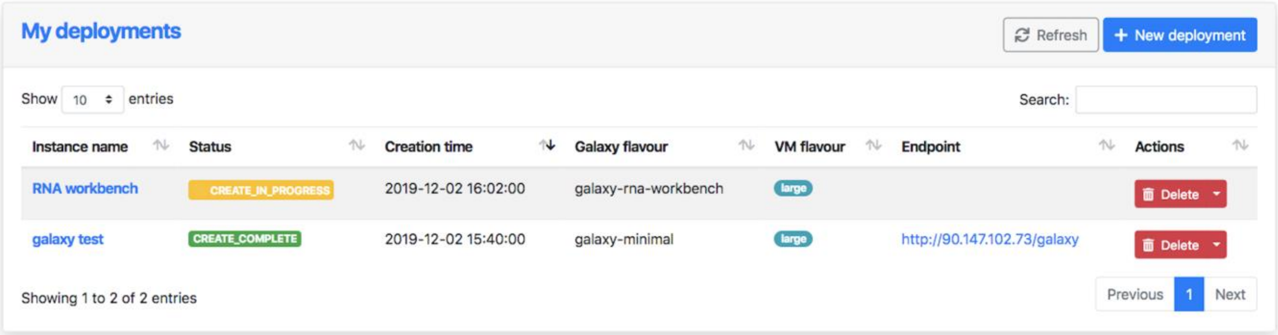

Fig 4. Laniakea Dashboard info and management interface. It reports the name, current status, creation time, initial Galaxy flavour, virtual hardware setup (VM flavour), and the URL (Endpoint) of each Galaxy instance generated by the user. Galaxy instances that are needed no more can be deleted using the "Delete" button.

When the Galaxy deployment procedure terminates, the user is notified with an e-mail message, and a new entry is added to the "My Deployments" page of the Laniakea Dashboard (Fig 4). The user retains full administrator rights over each created instance and can control and customise them (e.g., installing new tools, adding users, sharing datasets, setting quotas) using the standard Galaxy interface. Expert users that need access to the underlying virtual machine, e.g., to implement novel tools or to tweak advanced configuration options not available from the Galaxy administration interface, can do so using the SSH key they provided during the configuration.

Galaxy flavours

As with any other Galaxy instance, users of Laniakea can equip their personal Galaxy instance with a variety of tools that can be browsed, searched, retrieved, and automatically installed from Tools Sheds using the Galaxy admin interface. However, to lighten the effort required from users and to set them in working conditions in the shortest possible time, Laniakea provides also a handy set of four domain-specific "*Galaxy flavours*", that is Galaxy instances already configured with curated collections of tools covering some of the most common NGS analysis pipelines. The provided tools have been extensively tested, organised in workflows, and are ready to be used out-of-the-box.

Apart from the basic "minimal" flavour, providing the standard set of default tools embedded in any Galaxy installation, the four available Galaxy flavours are: "Epigen", "RNA workbench", "GDC Somatic Variant Calling" and "CoVaCS". The "Epigen" flavour is based upon the layout of Epigen Project Galaxy server (http://www.beaconlab.it/epigalaxy) and provides a selection of tools for the analysis of ChIP-Seq and RNA-Seq data. The "RNA workbench" flavour is based on [37] and includes more than 50 tools dedicated to RNA-centric analyses including, e.g., alignment, annotation, secondary structure profiling, target prediction. The "GDC Somatic Variant Calling" flavour (Fig 5) is a porting of the Genomic Data Commons (GDC) pipeline for the identification of somatic variants on whole exome/genome sequencing data (https://gdc.cancer.gov/node/246). Finally, the "CoVaCS" flavour implements the homonymous workflow (Fig. S2) described in [38], and comprehend a set of tools for genotyping and variant annotation of whole genome/exome and target-gene sequencing data.

New flavours can be implemented easily in Laniakea providing the corresponding virtual machine snapshot, Docker container, and Ansible recipe needed for the corresponding deployment strategies (Express, Docker, and Live Build). A simple and documented procedure, requiring only the list of desired Galaxy tools, is in place to allow users to design customized flavours to be made available in Laniakea.

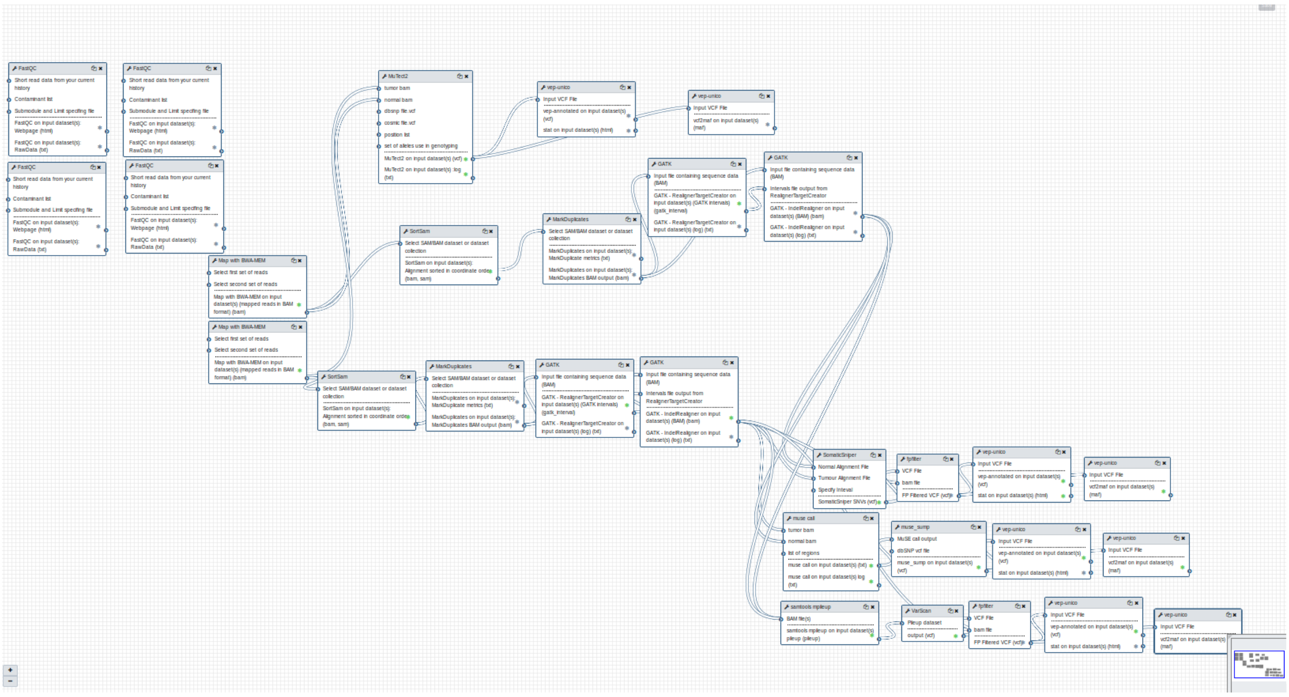

Fig. 5. The GDC Somatic Variant Calling analysis pipeline implemented as a Galaxy workflow for the corresponding flavour. The workflow design interface of Galaxy is a powerful instrument to elaborate complex workflows chaining together the output and the input of different tools in an intuitive fashion.

Reference data volume

To avoid useless replication of reference data and facilitate the reproducibility of analyses, Laniakea supports linking Galaxy to read-only CVMFS volumes that can be shared among multiple instances. As proof of concept, the Laniakea@ReCaS service (see “Laniakea@ReCaS testing and production service”) currently provides access to three different reference data repositories. The first, named “ELIXIR-IT Galaxy reference data”, is a basic, manually curated, reference dataset containing the latest releases of the human, mouse, yeast, fruit fly and *A. thaliana* genomes and the corresponding indexes for Bowtie [39], Bowtie2 [40] and BWA [41,42]. The second, named “ELIXIR-IT Galaxy CoVaCS reference data”, is a repository tailored for variant calling in human, to be used with the CoVaCS flavour, with a collection of publicly available datasets of human genetic variants derived from a selection of large-scale resequencing projects [43–45], along with curated human genome assemblies and indexes obtained from the GATK bundle repository [46]. Finally, a mirror of the Galaxy project “by hand” reference repository, named “usegalaxy.org Galaxy reference data”, is provided.

Data volume encryption

Unless proper countermeasures are in place, data stored on a data volume linked to a virtual machine can potentially be exposed to anyone with legitimate or illegitimate access to the underlying IaaS and physical hardware [47]. This

consideration may result in technical, ethical and legal concerns, in particular when sensible human genetic data are involved. These considerations are exceptionally relevant for health operators and researchers involved in clinical bioinformatics or with the analysis of sensible data in general. We tackle this issue by providing Laniakea users with the option to attach to any Galaxy instance a secure data storage volume with file system level encryption. The storage volume is encrypted using a *key stretching* approach: a randomly generated master key is encrypted using a instance-specific passphrase through PBKDF2 key derivation. This approach makes both brute force and *rainbow tables* [48] based attacks more computationally expensive, and at the same time allows for multiple passphrases and passphrase change or revocation without re-encryption. To make the system even more robust and prevent users from using weak passphrases and/or losing them, a randomly generated alphanumerical key is univocally assigned to each user and safely stored by Laniakea using Hashicorp Vault (https://www.vaultproject.io/). In this way, the passphrase can, at the same time, be seen and used only by its legitimate holder without the need to actually remember it. Finally, the LUKS *anti-forensic splitter* feature protects data against recovery attempts after volume deletion. The resulting layout consists of Galaxy running transparently on top of the encrypted volume (Fig, 6). This means that Galaxy instance attached to the encrypted volume retains for its users all the functionalities, data sharing included, of any other Galaxy instance without filesystem level encryption.

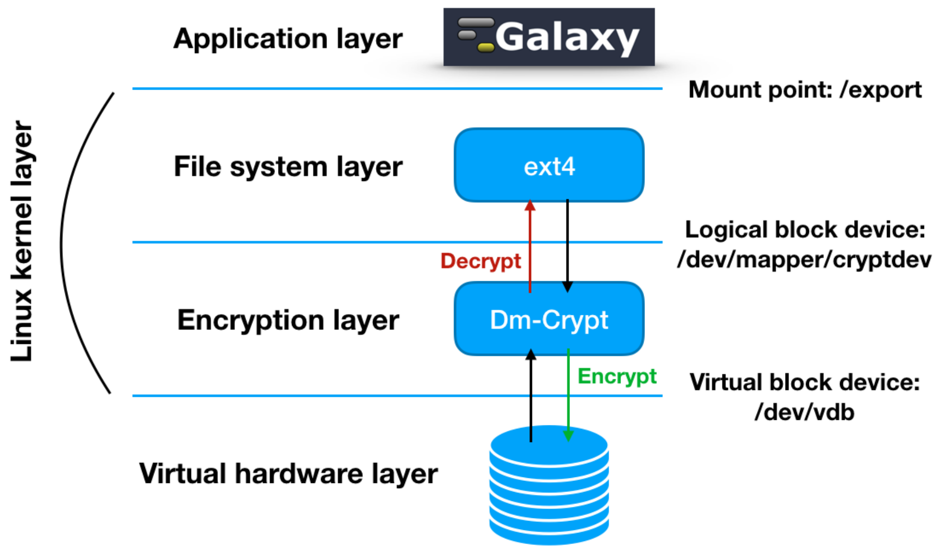

Fig. 6. The relationship between Galaxy, the file system, and dm-crypt. Data are encrypted and decrypted on-the-fly when writing and reading through dm-crypt. The underlying disk encryption layer is completely transparent to Galaxy.

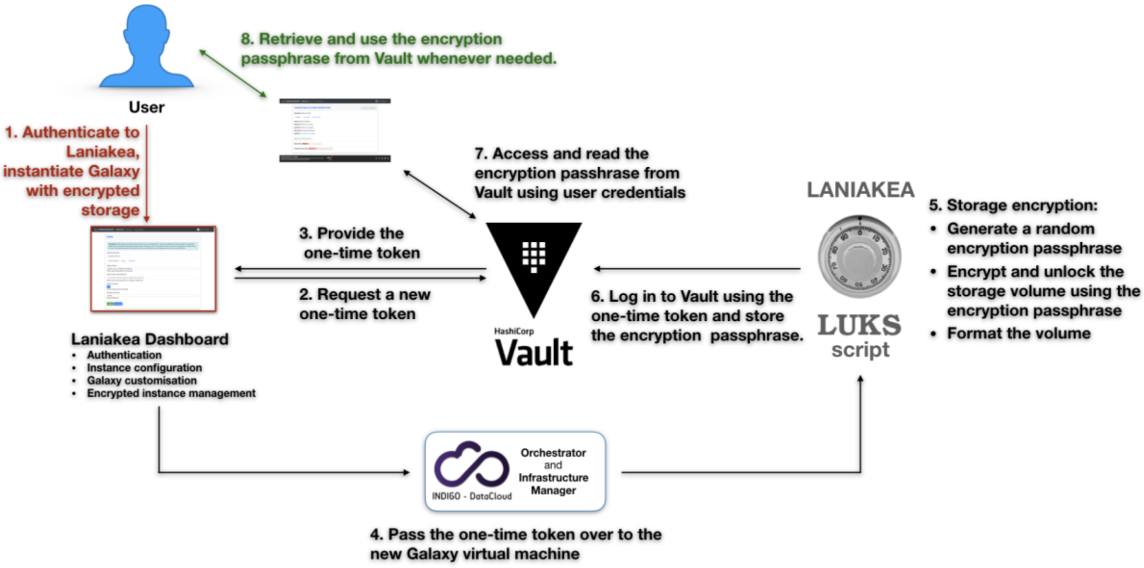

Fig. 7. Storage encryption workflow. (1) The user logs into the Laniakea Dashboard and enables data encryption when configuring a new Galaxy instance. (2-3) The Dashboard contacts Hashicorp Vault, using a IAM token, to retrieve a one-time Vault token. The one-time token is used to avoid transmitting user credentials over the infrastructure and limit any potential damage from a malicious attacker intercepting it (4) The one-time token is passed to the encryption script on the virtual machine through the INDIGO Orchestrator service. (5) A random passphrase is generated, and the data volume is encrypted by LUKS, unlocked and formatted. It is now ready to be attached to the new Galaxy instance. (6) The encryption script logs in to Vault using the one-time token and stores the encryption passphrase that will be accessible only by the Laniakea user that requested the encrypted volume (7-8) The user can retrieve the passphrase at any time using the Dashboard. For example, if the encrypted volume needs to be re-mounted (usually after a reboot of the Galaxy virtual machine) the user can retrieve the passphrase from Vault and unlock the volume using the Dashboard.

The encryption procedure (Fig. 7) is completely automated and can be enabled by the user through the Laniakea Dashboard during the configuration of a new Galaxy instance. A similar procedure allows the user to re-mount the data volume directly from the Dashboard when and if an encrypted Galaxy instance is rebooted.

To validate the data encryption strategy, we simulated two different attack scenarios. In the first scenario, the attacker obtains unauthorized access to the unmounted encrypted volume, while the second simulates the improper use of administrator IaaS privileges when the LUKS volume is already unlocked and in use by a running Galaxy instance.

In the first scenario, we compared two identical volumes, one encrypted and the other not, both attached to the same Galaxy instance, with the same set of permissions and each containing a copy of the same plain text file. Once detached, we created a binary image file of each volume and tried to access the data structure through *hex dump*. We were able

to quickly retrieve the original content of the text file from the non-encrypted volume while the hex dump of the encrypted volume did not contain the original text in any discernible form. In the second scenario, we tried to read data from the volume already mounted on a running Galaxy instance using the OpenStack cloud controller. We were not able to gain access to the LUKS encrypted device by any means without providing the correct passphrase.

This approach should go a long way to reasonably insulate any data uploaded to an encrypted Laniakea Galaxy instance from malicious access as long as the Galaxy instance itself and the encryption passphrase remain uncompromised.

Cluster support

For an IaaS administrator, the option to offer static or elastic cluster support to users provides the alternative between guaranteeing a constant pool of resources to those instances attached to a static cluster, or greater control over the efficient usage of the available resources, for those instances that instead rely on an elastic cluster. The INDIGO PaaS layer used by Laniakea supports both solutions and can deploy static or elastic clusters on top of existing OpenStack/OpenNebula, Amazon AWS, and Google Cloud. Elastic clusters (Fig. 8) dynamically scale the number of nodes available to a Galaxy instance depending on its workload. From the user point of view, both solutions enable straightforward access to computational resources beyond those assigned to single node Galaxy instances, enabling a higher number of simultaneous Galaxy users, quicker execution of jobs, and additional room for computationally intensive analyses.

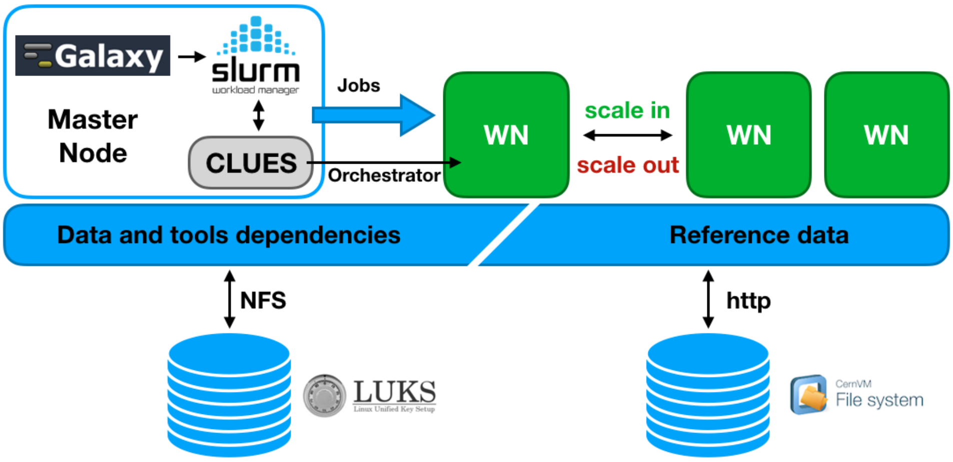

Fig. 8. Galaxy elastic cluster architecture. Initially, only the master node, that hosts Galaxy, SLURM, and CLUES, is deployed. The SLURM queue is monitored by CLUES, and new worker nodes are deployed to process pending jobs up to the maximum number set during cluster configuration, thus adapting resources availability to the current workload. The user home

directory and persistent storage are shared among master and worker nodes through the Network File System (NFS) enabling the sharing of CONDA tools dependencies. The CVMFS shared volume is also mounted on each worker node so to ensure that tools have access to reference data.

Laniakea@ReCaS testing and production service

We ran a testing programme for the prototypal Laniakea service, named Laniakea@ReCaS, starting on Dec 2018 and ending on Nov 2019. 128 CPUs, 256 GB of RAM, and 10 TB of disk storage were reserved for the service at the ReCaS cloud facility (https://www.recas-bari.it/index.php/en/). The programme involved the participation of fifteen users from several research institutions and scientific backgrounds that were either directly invited by us or asked to join the programme after being introduced to Laniakea during workshops or other dissemination events. The users were requested to stress-test the service by deploying, deleting, and extensively using one or more virtual Galaxy instances for their daily research activities. During this programme, we collected feedback from users and worked in fixing the juvenile issues of the service, also prioritizing a list of features for future development.

Access to the production service will be offered shortly to researchers and other stakeholders on a per-project basis through an open-ended call in a fashion similar to the ongoing ELIXIR-IT HPC@CINECA service (submitted and under review). In brief, each project proposal will be evaluated by a scientific and technical board. Successful proposals will be granted a standard package of computational resources to be used with Laniakea for an amount of time compatible with the project requirements.

Discussion and Conclusions

Laniakea provides a solution to easily include a Galaxy on-demand service within the portfolio of public and private scientific cloud providers. This result is achieved by leveraging the INDIGO middleware that, having been designed to support a vast array of scientific services, is extensively used and supported across several European cloud infrastructures. Furthermore, support for federated cloud infrastructures allows piloting computational resources from remote INDIGO services, bypassing the need to have the INDIGO software stack installed on each member of the federated cloud infrastructure.

Laniakea supports a variety of Galaxy setups and deployment strategies that can be useful in multiple employment scenarios, e.g., small research groups, developers, didactic purposes, and even production-grade instances with static

or elastic cluster support enabling multiple concurrent users and computationally demanding analyses. Helping in bypassing the need to host and maintain local hardware and software infrastructures in those scenarios, Laniakea favours a more efficient use of the available resources, harnessing the improved reliability offered by cloud environments and enhancing reproducibility of bioinformatics analyses through Galaxy.

New Laniakea Galaxy flavours can be quickly developed and shared in the form of VM snapshots, Docker containers, or Ansible recipes. These pre-configured Galaxy instances save users from lengthy routines of tools installation and provide a mean to nimbly develop and make available data analysis pipelines as we did with the CoVaCS and GDC Somatic Variant flavours. In fact, domain-specific public Galaxy instances have already been developed using Laniakea as a platform to implement and put into production novel services (see [49] and www.beaconlab.it/VINYL, currently under review). At the same time, Laniakea users keep the ability to customise their Galaxy instances with all the options and instruments commonly available to any other Galaxy admin: e.g., Tool Sheds, user quotas, roles, groups, data libraries, jobs managing, API keys, etc.. Finally, the data security layer of Laniakea represents a significant step in the direction of addressing the common issue posed by the analysis of sensitive data in public cloud infrastructures.

Future development directions for Laniakea will aim at improving the compatibility of Laniakea with a broader array of existing cloud setups, at extending cluster support to other resource managers (e.g., TORQUE [50], HTCondor [51], etc...) and at widening the selection of tools to be made available on-demand beyond Galaxy.

Availability and requirements

The Laniakea web portal is available at https://laniakea-elixir-it.github.io. Source code and service configuration files are hosted on github at https://github.com/Laniakea-elixir-it. Laniakea ansible roles, being part of INDIGO source code, are hosted on the INDIGO git repository at https://github.com/indigo-dc.

Complete documentation for Laniakea, comprising a step-by-step guide of Laniakea installation, is available at https://laniakea.readthedocs.io/en/latest/

Laniakea docker containers are hosted on DockerHub at https://cloud.docker.com/u/laniakeacloud/repository/list .

All software developed for Laniakea is licensed under GPLv3, with the exception of ansible roles that, being part of the INDIGO software catalogue, are released under Apache-2.0 license.

Operating system: Platform independent

Programming language: JavaScript, Python, Shell, XML, YAML

Other requirements: Linux, Docker, INDIGO-Datacloud PaaS services, INDIGO IAM, Ansible.

Methods

INDIGO-DataCloud middleware

Laniakea builds on the INDIGO software catalogue [32] (https://github.com/indigo-dc). In particular:

- The Identity and Access Management (IAM) is an Authentication and Authorisation Infrastructure (AAI) service that manages user identities, attributes (e.g., affiliation and groups membership) and authorization policies to access federated PaaS and manage heterogeneous and distributed resources through a single user account.
- The INDIGO PaaS [52] serves as the abstraction layer for the definition and provision of the resources required by users, managing the transparent deployment of virtual machines on OpenStack (https://www.openstack.org), OpenNebula (https://opennebula.org) and commercial cloud

providers. INDIGO PaaS processes requests in the form of TOSCA automation templates. These are modular documents that use YAML syntax and Ansible roles to describe the properties and configuration of the virtual hardware and software components, and the sequence of actions needed to achieve the deployment of a virtual environment.

- CLUES [53] is an elasticity manager for HPC clusters that enables dynamic cluster resources scaling, deploying and powering-on new working nodes depending on the current workload of the cluster and powering-off and deleting them when they are no longer required.

We developed the set of TOSCA templates (https://github.com/indigo-dc/tosca-types/blob/master/custom_types.yaml) required to automate the installation and configuration of software and virtual hardware for Laniakea Galaxy instances (https://github.com/indigo-dc/tosca-templates , https://github.com/Laniakea-elixir-it/laniakea-dashboard-config).

The Galaxy environment

- Galaxy production environment standard software stack: CentOS 7, PostgreSQL, Nginx, uWSGI and ProFTPD. Apart from the operative system, for which there are no official recommendations, this configuration is rooted in the guidelines for production environments issued by Galaxy Project (https://docs.galaxyproject.org/en/latest/admin/production.html). We implemented the Ansible role required to orchestrate the automatic installation and configuration of the environment (https://github.com/indigo-dc/ansible-role-galaxycloud).
- Tools: Laniakea employs the official Galaxy Project library Ephemeris (https://github.com/galaxyproject/ephemeris) to manage Galaxy tools installation. We developed an ansible role (https://github.com/indigo-dc/ansible-role-galaxycloud-tools) to use Ephemeris with Laniakea and the YAML recipes corresponding to the Galaxy flavours (https://github.com/indigo-dc/Galaxy-flavors-recipes) currently available in Laniakea. Several wrappers have been developed for the GDC Somatic Variant Calling and CoVaCS Galaxy flavours and made available on the official Galaxy Test Tool Shed at (https://tinyurl.com/elixir-it-tts).

- CernVMFile System (CVMFS) [54] is used by Laniakea to provide reference data to multiple Galaxy instances. Galaxy instances can both be linked to and use the official usegalaxy.org CVMFS repository (https://galaxyproject.org/admin/reference-data-repo/) or use any other custom CVMFS repository. As a proof of concept, we set up a local CVMFS repository for those tools in the GDC Somatic Variant Calling and CoVaCS flavours requiring reference data not available in the main repository. We did automatize both the configuration of Galaxy to make use of a CVMFS server (https://github.com/indigo-dc/ansible-role-galaxycloud-refdata) and the creation of new CVMFS repositories on the IaaS (https://github.com/indigo-dc/ansible-role-cvmfs-server and https://github.com/indigo-dc/ansible-role-cvmfs-client).

Finally, the galaxyctl python API (https://github.com/Laniakea-elixir-it/galaxyctl) has been developed to manage and monitor the status of Galaxy instances from the dashboard of Laniakea.

Docker containers

Galaxy dockers used by Laniakea are based on the official Galaxy Docker port (http://bgruening.github.io/docker-galaxy-stable/). We developed an ansible role (https://github.com/indigo-dc/ansible-role-galaxycloud-docker) to modify the official Galaxy Docker container and make readily available Laniakea Galaxy flavours also through this medium. All Laniakea Docker containers are available on DockerHub (https://hub.docker.com/u/laniakeacloud)

Storage encryption

The encryption layer is based on LUKS (Linux Unified Key Setup) [55], the current standard for encryption on Linux platforms. It provides robustness against low-entropy passphrase attacks using salting and iterated PBKDF2 passphrase hashing. LUKS supports secure management for multiple user passwords, allowing to add, change and revoke passwords without re-encryption of the whole device. We developed a bash script (https://github.com/Laniakea-elixir-it/fast-luks) to perform storage encryption in Laniakea, an ansible role (https://github.com/indigo-dc/ansible-role-galaxycloud-os) to automate the whole procedure, and the the luksctl python package (https://github.com/Laniakea-elixir-it/luksctl) and API (https://github.com/Laniakea-elixir-it/luksctl_api) to let users easily create and manage encrypted volumes from the Dashboard.

Hashicorp Vault

To let users securely store and access encryption passphrases and SSH private keys Laniakea relies on Hashicorp Vault (https://www.vaultproject.io/) secrets management software. Data stored on Vault are encrypted with 256 bit AES

(Advanced Encryption Standard) cipher in the Galois Counter Mode (GCM) with a randomly generated nonce. The Laniakea's Hashicorp Vault configuration is available at https://github.com/Laniakea-elixir-it/vault_config.

Laniakea dashboard

Laniakea dashboard is based on the Orchestrator Dashboard developed in the framework of the DEEP-HybridDataCloud H2020 project (https://deep-hybrid-datacloud.eu/), using the Flask web micro-framework (https://flask.palletsprojects.com/en/1.1.x/) and the Bootstrap 4 toolkit (https://bootstrapcreative.com/shop/bootstrap-4-toolkit/). Laniakea dashboard code is available at (https://github.com/Laniakea-elixir-it/orchestrator-dashboard/tree/laniakea-stable).

Acknowledgements

This work has been supported by the European Commission H2020 research and innovation program under grant agreement RIA 653549.

We thank Davide Corà and Francesco Favero (Dept. of Translational Medicine, Piemonte Orientale University), Gianmauro Cuccuru (Dept. of Computer Science, University of Freiburg), Emanuele Bonetti (Istituto Ortopedico Rizzoli), Antonio Parisi (Istituto Zooprofilattico Sperimentale della Puglia e della Basilicata), Martina Alverà (Dept. of Biosciences, University of Milan), Davide Cangelosi (Laboratory of Molecular Biology, IRCCS Istituto Giannina Gaslini), Angelo Romano (Istituto Zooprofilattico Sperimentale Piemonte, Liguria e Valle d'Aosta), Rossano Atzeni (CRS4 Centro di Ricerca, Sviluppo e Studi Superiori in Sardegna), Rick Jansen, Saskia Hiltemann, and Andrew Stubbs (Erasmus Medical Centre, Rotterdam, The Netherlands) for their kind and precious assistance with Laniakea testing.

Competing interests

The authors declare that they have no competing interests

References

1. Attwood TK, Blackford S, Brazas MD, Davies A, Schneider MV. A global perspective on evolving bioinformatics and data science training needs. Brief Bioinform. 2019;20:398–404.
2. Via A, Attwood TK, Fernandes PL, Morgan SL, Schneider MV, Palagi PM, et al. A new pan-European Train-the-Trainer programme for bioinformatics: Pilot results on feasibility, utility and sustainability of learning. Brief Bioinform. 2019;20:405–15.
3. McGrath A, Champ K, Shang CA, van Dam E, Brooksbank C, Morgan SL. From trainees to trainers

to instructors: Sustainably building a national capacity in bioinformatics training. PLoS Comput Biol. 2019;15:1–12.
4. Piccolo SR, Frampton MB. Tools and techniques for computational reproducibility. Gigascience. 2016;5:1–13.
5. Kumar S, Dudley J. Bioinformatics software for biologists in the genomics era. Bioinformatics. 2007;23:1713–7.
6. Attwood TK, Blackford S, Brazas MD, Davies A, Schneider MV. A global perspective on evolving bioinformatics and data science training needs. Brief Bioinform [Internet]. 2017;1–7. Available from: http://academic.oup.com/bib/article/doi/10.1093/bib/bbx100/4096809/A-global-perspective-on-evolving-bioinformatics
7. Beckmann JS, Lew D. Reconciling evidence-based medicine and precision medicine in the era of big data: Challenges and opportunities. Genome Med [Internet]. Genome Medicine; 2016;8:1–11. Available from: http://dx.doi.org/10.1186/s13073-016-0388-7
8. Cohen-Boulakia S, Belhajjame K, Collin O, Chopard J, Froidevaux C, Gaignard A, et al. Scientific workflows for computational reproducibility in the life sciences: Status, challenges and opportunities. Futur Gener Comput Syst [Internet]. Elsevier B.V.; 2017;75:284–98. Available from: http://dx.doi.org/10.1016/j.future.2017.01.012
9. Afgan E, Baker D, Batut B, van den Beek M, Bouvier D, Čech M, et al. The Galaxy platform for accessible, reproducible and collaborative biomedical analyses: 2018 update. Nucleic Acids Res [Internet]. 2018;46:W537–44. Available from: http://dx.doi.org/10.1093/nar/gky379
10. Euan A. Towards precision medicine. Nat Rev Genet. 2016;17.
11. Lightbody G, Haberland V, Browne F, Taggart L, Zheng H, Parkes E, et al. Review of applications of high-throughput sequencing in personalized medicine: barriers and facilitators of future progress in research and clinical application. Brief Bioinform. 2018;1–17.
12. Batut B, Hiltemann S, Bagnacani A, Baker D, Bhardwaj V, Blank C, et al. Community-Driven Data Analysis Training for Biology. Cell Syst. 2018;6:752-758.e1.
13. Mell P, Grance T. The NIST Definition of Cloud Computing Recommendations of the National Institute of Standards and Technology. Nist Spec Publ. 2011;145:7.
14. Langmead B, Nellore A. Cloud computing for genomic data analysis and collaboration. Nat Rev Genet [Internet]. Nature Publishing Group; 2018;19:208–19. Available from: http://www.nature.com/doifinder/10.1038/nrg.2017.113
15. Karim R, Michel A, Zappa A, Baranov P, Sahay R, Rebholz-schuhmann D. Improving data workflow systems with cloud services and use of open data for bioinformatics research. Brief Bioinform [Internet]. 2017;1–16. Available from: http://fdslive.oup.com/www.oup.com/pdf/production_in_progress.pdf
16. Pavlovich M. Computing in Biotechnology: Omics and Beyond. Trends Biotechnol [Internet]. Elsevier Ltd; 2017;35:479–80. Available from: http://dx.doi.org/10.1016/j.tibtech.2017.03.011
17. Warth B, Levin N, Rinehart D, Teijaro J, Benton HP, Siuzdak G. Metabolizing Data in the Cloud. Trends Biotechnol [Internet]. Elsevier Ltd; 2017;35:481–3. Available from: http://dx.doi.org/10.1016/j.tibtech.2016.12. 010
18. Emami Khoonsari P, Moreno P, Bergmann S, Burman J, Capuccini M, Carone M, et al. Interoperable and scalable data analysis with microservices: Applications in metabolomics. Bioinformatics. 2019;35:3752–60.
19. Verderame L, Merelli I, Morganti L, Corni E, Cesini D, D'Agostino D, et al. A secure cloud-edges computing architecture for metagenomics analysis. Futur Gener Comput Syst [Internet]. Elsevier B.V.; 2019; Available from: https://doi.org/10.1016/j.future.2019.09.013
20. Griebel L, Prokosch HU, Köpcke F, Toddenroth D, Christoph J, Leb I, et al. A scoping review of cloud computing in healthcare. BMC Med Inform Decis Mak. 2015;15:1–16.

21. Bellazzi R. Big Data and Biomedical Informatics : A Challenging Opportunity Big Data : Why Bother ? Big Data : Must-have or. Yearb Med Inform. 2014;9:8–13.
22. Liu B, Madduri RK, Sotomayor B, Chard K, Lacinski L, Dave UJ, et al. Cloud-based bioinformatics workflow platform for large-scale next-generation sequencing analyses. J Biomed Inform [Internet]. Elsevier Inc.; 2014;49:119–33. Available from: http://dx.doi.org/10.1016/j.jbi.2014.01.005
23. Afgan E, Baker D, Coraor N, Chapman B, Nekrutenko A, Taylor J. Galaxy CloudMan: Delivering cloud compute clusters. BMC Bioinformatics. 2010;11:2–7.
24. Afgan E, Chapman B, Taylor J. CloudMan as a platform for tool, data, and analysis distribution. BMC Bioinformatics [Internet]. BMC Bioinformatics; 2012;13:1. Available from: BMC Bioinformatics
25. Afgan E, Sloggett C, Goonasekera N, Makunin I, Benson D, Crowe M, et al. Genomics Virtual Laboratory: A practical bioinformatics workbench for the cloud. PLoS One. 2015;10:1–20.
26. Peters K, Bradbury J, Bergmann S, Capuccini M, Atauri P De, Ebbels TMD, et al. PhenoMeNal : Processing and analysis of Metabolomics data in the Cloud. 2018;
27. Krieger MT, Torreno O, Trelles O, Kranzlmüller D. Building an open source cloud environment with auto-scaling resources for executing bioinformatics and biomedical workflows. Futur Gener Comput Syst [Internet]. Elsevier B.V.; 2017;67:329–40. Available from: http://dx.doi.org/10.1016/j.future.2016.02.008
28. Afgan E, Baker D, van den Beek M, Blankenberg D, Bouvier D, Čech M, et al. The Galaxy platform for accessible, reproducible and collaborative biomedical analyses: 2016 update. Nucleic Acids Res. 2016;44:W3–10.
29. Afgan E, Baker D, Coraor N, Goto H, Paul IM, Makova KD, et al. Harnessing cloud computing with Galaxy Cloud. Nat Biotechnol. 2011;29:972–4.
30. Caballer M, Zala S, García ÁL, Moltó G, Fernández PO, Velten M. Orchestrating Complex Application Architectures in Heterogeneous Clouds. J Grid Comput. 2017;1–16.
31. Attardi G, DI Martino B, Esposito A, Mastroianni M. Using federated cloud platform to implement academia services for research and administration. Proc - 32nd IEEE Int Conf Adv Inf Netw Appl Work WAINA 2018. IEEE; 2018;2018-Janua:413–8.
32. Campos DSI, Marco LGJ, Solagna DLP, Matyska JGL, Hardt PFM, Dutka GDL, et al. INDIGO-DataCloud : a Platform to Facilitate Seamless Access to E-Infrastructures. J Grid Comput [Internet]. 2018; Available from: https://link.springer.com/article/10.1007%2Fs10723-018-9453-3
33. Salomoni D, Campos I, Gaido L, Donvito G, Antonacci M, Fuhrman P, et al. INDIGO-Datacloud: foundations and architectural description of a Platform as a Service oriented to scientific computing. 2016;1–31. Available from: http://arxiv.org/abs/1603.09536
34. Merkel D. Docker: lightweight Linux containers for consistent development and deployment. Linux J [Internet]. 2014;2014:2. Available from: http://dl.acm.org/ft_gateway.cfm?id=2600241&type=html%5Cnhttp://www.linuxjournal.com/content/docker-lightweight-linux-containers-consistent-development-and-deployment
35. Ceccanti A, Hardt M, Wegh B, Millar AP, Caberletti M, Vianello E, et al. The INDIGO-Datacloud Authentication and Authorization Infrastructure. J Phys Conf Ser. 2017;898.
36. Linden M, Prochazka M, Lappalainen I, Bucik D, Vyskocil P, Kuba M, et al. Common elixir service for researcher authentication and authorisation [version 1; referees: 3 approved, 1 approved with reservations]. F1000Research. 2018;7.
37. Grüning BA, Fallmann J, Yusuf D, Will S, Erxleben A, Eggenhofer F, et al. The RNA workbench: Best practices for RNA and high-throughput sequencing bioinformatics in Galaxy. Nucleic Acids Res. 2017;45:W560–6.
38. Chiara M, Gioiosa S, Chillemi G, D'Antonio M, Flati T, Picardi E, et al. CoVaCS: a consensus

variant calling system. BMC Genomics [Internet]. BMC Genomics; 2018;19:120. Available from: https://bmcgenomics.biomedcentral.com/articles/10.1186/s12864-018-4508-1
39. Langmead B, Trapnell C, Pop M, Salzberg SL. Ultrafast and memory-efficient alignment of short DNA sequences to the human genome. Genome Biol. 2009;10.
40. Langmead B, Salzberg SL. Fast gapped-read alignment with Bowtie 2. Nat Methods. 2012;9:357–9.
41. Li H, Li H, Durbin R, Durbin R. Fast and accurate short read alignment with Burrows-Wheeler transform. Bioinformatics [Internet]. 2009;25:1754–60. Available from: http://www.pubmedcentral.nih.gov/articlerender.fcgi?artid=2705234%5C&tool=pmcentrez%5C&rendertype=abstract%5Cnpapers2://publication/doi/10.1093/bioinformatics/btp324
42. Li H, Durbin R. Fast and accurate long-read alignment with Burrows-Wheeler transform. Bioinformatics. 2010;26:589–95.
43. Sherry ST. dbSNP: the NCBI database of genetic variation. Nucleic Acids Res [Internet]. 2001;29:308–11. Available from: https://academic.oup.com/nar/article-lookup/doi/10.1093/nar/29.1.308
44. Mills RE, Pittard WS, Mullaney JM, Farooq U, Creasy TH, Mahurkar AA, et al. Natural genetic variation caused by small insertions and deletions in the human genome. Genome Res. 2011;21:830–9.
45. Auton A, Abecasis GR, Altshuler DM, Durbin RM, Bentley DR, Chakravarti A, et al. A global reference for human genetic variation. Nature. 2015;526:68–74.
46. McKenna A, Hanna M, Banks E, Sivachenko A, Cibulskis K, Kernytsky A, et al. The Genome Analysis Toolkit: a MapReduce framework for analyzing next-generation DNA sequencing data. Genome Res. Cold Spring Harbor Lab; 2010;
47. Yuchi X, Shetty S. Enabling security-aware virtual machine placement in IaaS clouds. Proc - IEEE Mil Commun Conf MILCOM. 2015;2015-Decem:1554–9.
48. Oechslin P. Making a Faster Cryptanalytic Time-Memory Trade-Off. 2003;617–30. Available from: http://link.springer.com/10.1007/978-3-540-45146-4_36
49. Zanardi N, Morini M, Tangaro MA, Zambelli F, Bosco MC, Varesio L, et al. PIPE-T : a new Galaxy tool for the analysis of RT-qPCR expression data. 2019;1–12.
50. Staples G. TORQUE Resource Manager. Proc 2006 ACM/IEEE Conf Supercomput [Internet]. New York, NY, USA: ACM; 2006. Available from: http://doi.acm.org/10.1145/1188455.1188464
51. Thain D, Tannenbaum T, Livny M. Distributed computing in practice: The Condor experience. Concurr Comput Pract Exp. 2005;17:323–56.
52. Caballer M, Blanquer I, Moltó G, de Alfonso C. Dynamic Management of Virtual Infrastructures. J Grid Comput. 2015;13:53–70.
53. de Alfonso C, Caballer M, Alvarruiz F, Hernández V. An energy management system for cluster infrastructures. Comput Electr Eng [Internet]. 2013;39:2579–90. Available from: https://linkinghub.elsevier.com/retrieve/pii/S0045790613001365
54. Buncic P, Aguado Sanchez C, Blomer J, Franco L, Harutyunian A, Mato P, et al. CernVM - A virtual software appliance for LHC applications. J Phys Conf Ser. 2010;219.
55. Fruhwirth C. New methods in hard disk encryption. Inst Comput Lang Theory Log … [Internet]. 2005; Available from: http://git.dyne.org/tomb/plain/doc/New_methods_in_HD_encryption.pdf

Supplementary table and figures

Click here to access/download
Supplementary Material
Laniakea_Supplementary.docx

Reply to reviewers of the original manuscript (see cover letter)

Click here to access/download

Supplementary Material

Laniakea Answer to Reviewers Comments.docx

Cover Letter Click here to download Personal Cover Cover_Letter_2.pdf

UNIVERSITÀ DEGLI STUDI DI MILANO
DIPARTIMENTO DI BIOSCIENZE

DBS Dipartimento di Bioscienze

Dear Editor,

Please find enclosed the resubmission of our manuscript **Laniakea: an open solution to provide Galaxy "on-demand" instances over heterogeneous cloud infrastructures** intended for the GigaScience Galaxy series.

We submitted the original version of the manuscript in December 2018 (manuscript number GIGA-D-18-00494) and received a "Reject" decision from the Editor, Scott Edmunds, following two "Major revision" recommendations from the reviewers.

After a detailed rebuttal letter, Scott consented to take a resubmission in his communication dated 20 March 2019, provided that the points raised by the referees were addressed.

We have now dedicated several months of work and much effort in improving Laniakea to address the remarks of the reviewers. We are now ready to resubmit the manuscript, which also has been extensively reworked, together with a detailed reply to the points raised by the reviewers.

Laniakea is now a mature technological product, and in fact, the Laniakea@ReCaS service features already several very active users. A second Laniakea service, dubbed Laniakea@GARR, has already hit the pre-production testing phase. Furthermore, Laniakea is being used as a platform to streamline the development and publishing of new Galaxy-based scientific tools, as mentioned in the revised manuscript.

We have employed these months also to improve the engagement of the Galaxy community, presenting Laniakea at numerous events, always gathering excellent feedback, and fostering collaboration opportunities. As a proof, Laniakea has been featured in some Galaxy News newsletters, the last one in December 2019 (https://galaxyproject.org/news/2019-12-galaxy-update/), while the Laniakea-based Galaxy server PIPE-T is featured in the January 2020 one (https://galaxyproject.org/news/2020-01-galaxy-update/).

We have prepared three anonymous accounts for the Laniakea@ReCaS that can be accessed by referees at https://laniakea-elixir-it.github.io/.

Username	Password
laniakea1	Laniakea_R001
laniakea2	Laniakea_R002
laniakea3	Laniakea_R003

Yours sincerely,
on behalf of all the authors

Federico Zambelli

Dipartimento di Bioscienze
Via Celoria, n°26 - CAP 20133, Italy
Tel 02 503 111- Fax 02 503 111
