# Supplementary material for: Laniakea: an open solution to provide Galaxy “on-demand” instances over heterogeneous cloud infrastructures
Source: Gigascience. 2020 Apr 6;9(4):giaa033. doi: 10.1093/gigascience/giaa033 (PMC7136032; doi:10.1093/gigascience/giaa033)
Supplement: giaa033_Laniakea_Supplementary [file giaa033_laniakea_supplementary.docx]

|  | **Laniakea / INDIGO** | **PhenoMeNal** | **Genomic Virtual Lab / CloudMan** |
| --- | --- | --- | --- |
| **Virtual cluster support** | SLURM | Kubernetes + KubeNow | SLURM + CloudMan |
| **Cluster elasticity** | SLURM + CLUES | - | SLURM + CloudMan |
| **Data protection** | Support to filesystem-level encryption | - | - |
| **Reference data sharing** | CernVM File System | - | CernVM File System |
| **Production-grade Galaxy instance (Nginx, PostgreSQL, proFTPd, uWSGI)** | Based on CENTOS 7 (supports also Ubuntu 16.04) | Based on Ubuntu 14.04 | Based Ubuntu 14.04/Ubuntu 16.04 |
| **Administrator access to Galaxy** | Http, ssh, ftp. | Http, ftp.  Ssh access to the kubernetes VM. Then, the administrator has to access the Galaxy Docker container. | Http, ssh, ftp. |
| **Galaxy instance build process** | VM Snapshot / Docker / Live build  The “Live build” option makes us of Ansible recipes to automatically install Galaxy over a bare CentOS image, retrieving the required software component from public online repositories. | Docker | VM Snapshot / Docker |
| **Installation of new Galaxy tools and**  **tools development** | Tools can be installed using Galaxy Tool Shed(s).  New tools can be developed and tested within Galaxy the usual way. | New tools have to be deployed on the kubernetes cluster using Docker containers. | Tools can be installed using Galaxy Tool Shed(s).  New tools can be developed and tested within Galaxy the usual way. |
| **Virtual technology** | Virtual machines / Docker | Docker | Virtual machines / Docker |
| **Authentication and Authorization** | INDIGO IAM and ELIXIR-AAI (both include support for Google Accounts). | ELIXIR-AAI (includes support for Google accounts) | Google / GitHub / Linkedin / Facebook accounts. |
| **Access to virtual resources** | Resources are transparently allocated from any cloud provider available to the user. When multiple providers are available, the IM select the most suitable based on current workloads and the amount of resources to be allocated. | The user can select among a set of supported cloud providers and provide the corresponding access credentials. | The user can select among a set of supported cloud providers and provide the corresponding access credentials. |
| **Supported Cloud Provider Managers** | INDIGO is open source and currently supports OpenStack, OpenNebula, Amazon AWS, MS Azure. | Terraform and kubernetes used to deploy Galaxy. Openstack, Amazon AWS, Google Cloud are supported. | Custom and open source solution, supports OpenStack and Amazon AWS. |
| **Cloud federation support** | INDIGO supports federation of multiple IaaS (private and public).  Policies can be put in place directing categories or groups of users to specific e-infrastructures. | The user needs to select the e-infrastructure where to deploy Galaxy. | The user needs to select the e-infrastructure where to deploy Galaxy. |
| **Deployment of the full software stack to local IaaS** | The INDIGO software stack, including Laniakea, can be installed on any compatible e-infrastructure. The procedure is documented. Ansible roles and Docker containers to automatically deploy each component are provided. | The Phenomenal portal is linked to the supported e-infrastructures.  The documentation to locally install the portal is available. | GVL can be installed on any compatible e-infrastructure or the portal can be linked to the supported e-infrastructure. The procedure is documented. |

**Table S1. Comparative features table of Laniakea, PhenoMeNal, and GVL. Virtual environment (light yellow background), Galaxy (light green background), and IaaS specific (light blue background) features are compared. Data for PhenoMeNal and GVL have been gathered from available documentation using our best effort. Only features related to the Galaxy on-demand service have been considered, both GVL and Phenomenal support the cloud deployment of additional software (e.g., RStudio, Jupyter).**

**
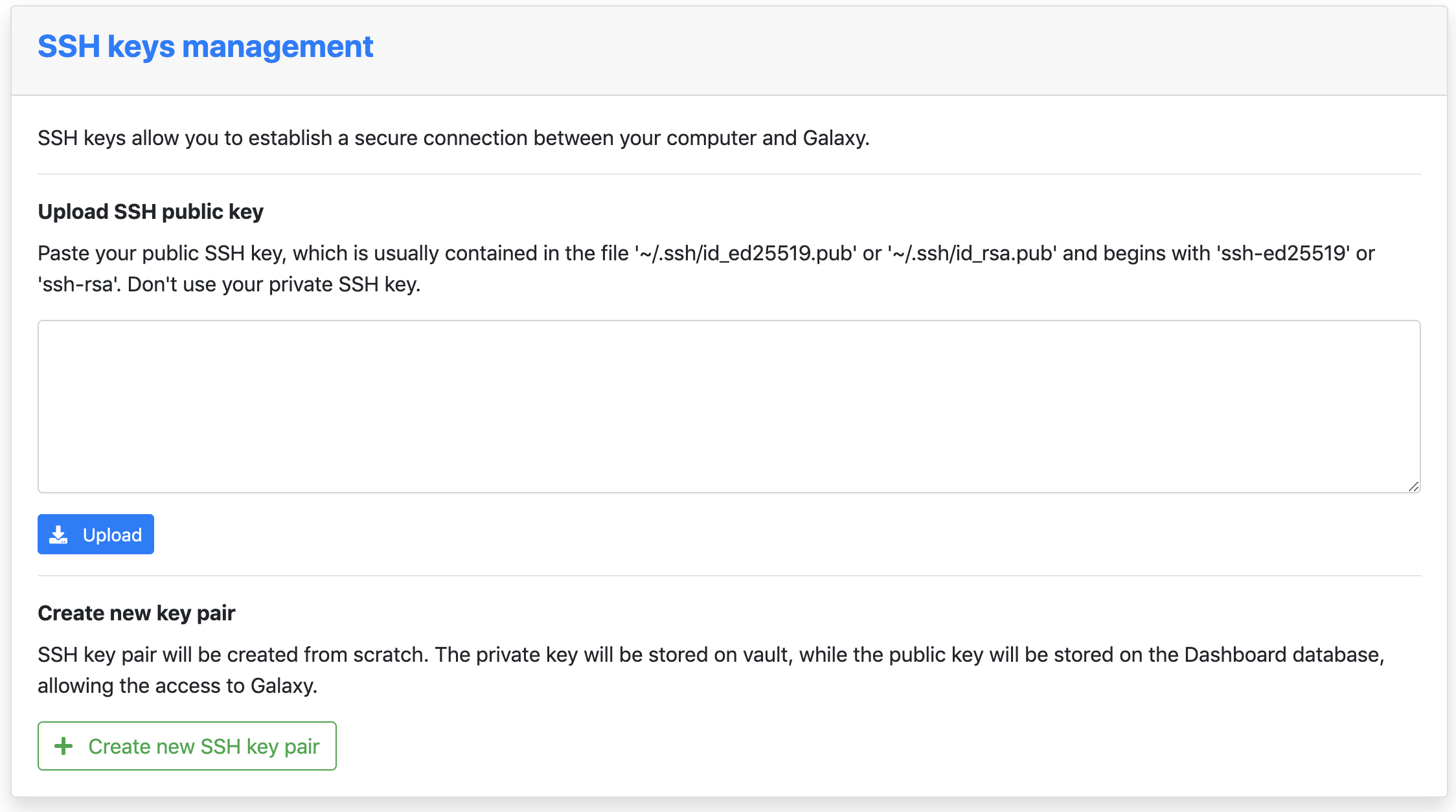
**

**Fig S1. Laniakea Dashboard SSH keys management. The interface allows to save the public SSH key of the user or to create a novel public/private key pair to be used within Laniakea. Private keys are safely stored using Hashicorp Vault (see Methods).**

**
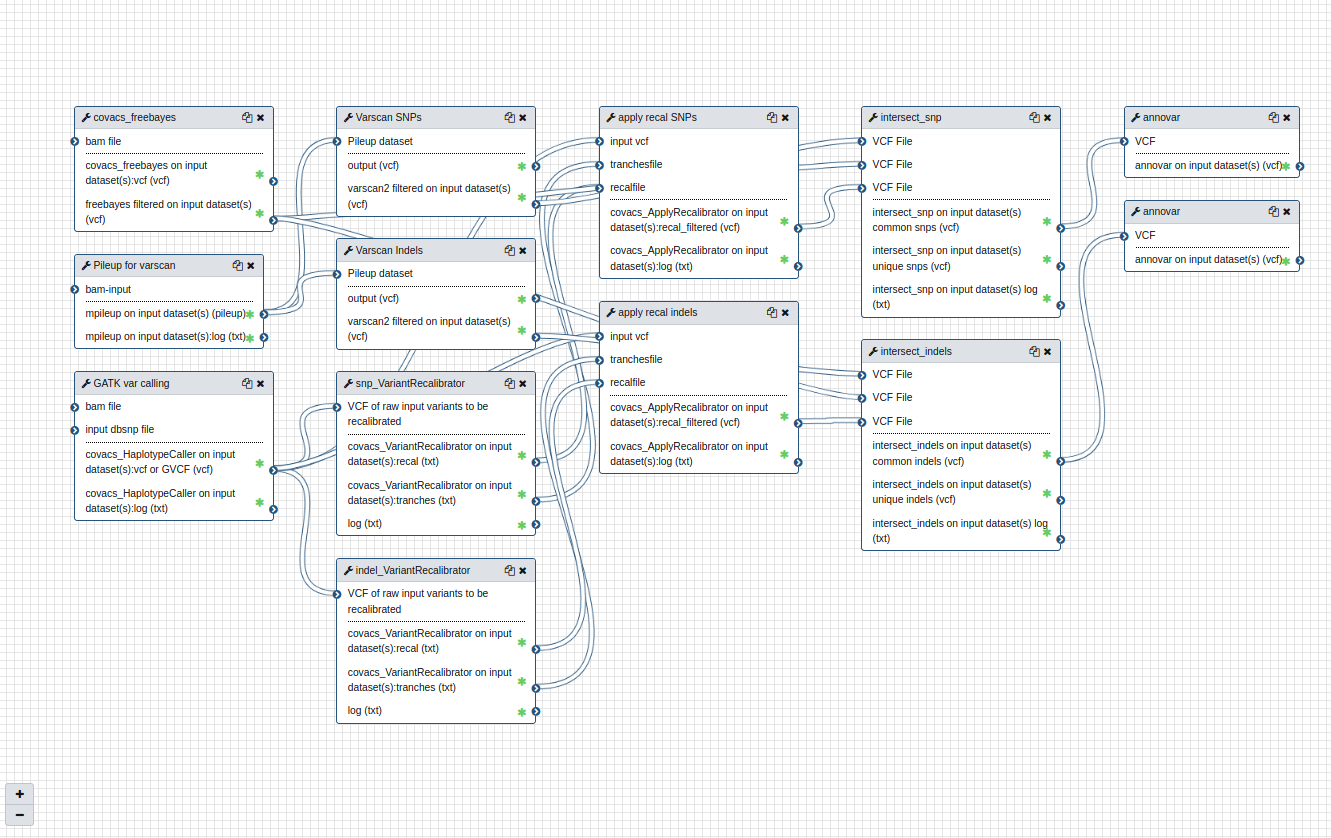
**

**Fig. S2. CoVaCS implemented as a Galaxy workflow for the corresponding flavour.**
