# Supplementary material for: Laniakea: an open solution to provide Galaxy “on-demand” instances over heterogeneous cloud infrastructures
Source: Gigascience. 2020 Apr 6;9(4):giaa033. doi: 10.1093/gigascience/giaa033 (PMC7136032; doi:10.1093/gigascience/giaa033)
Supplement: giaa033_Response-to-Reviewer_Comments_Original_Submission [file giaa033_response-to-reviewer_comments_original_submission.pdf]

Author's Response To Reviewer Comments

Close

Dear Editor,

I am pleased to submit to your attention the revision of our manuscript "Laniakea: an open solution to provide Galaxy “on-demand” instances over heterogeneous cloud infrastructures".

The manuscript has been given a careful and thorough language check. The comment of the referee has been addressed, adding the sentence "Galaxy jobs execution takes place within the deployed virtual environment." in the Overview section. We have slightly modified the "Laniakea@ReCaS testing and production service" section in order to reflect the current situation that has changed since submission, that is the Laniakea@ReCaS service is now in its production phase. It is now included in the EOSC Marketplace too. Several minor edits have been necessary in order to make the manuscript conform to the journal style.
I also included a version of the manuscript highlighting all the edits since the original submission.

Best regards.
Federico Zambelli

Close
