# Supplementary material for: Laniakea: an open solution to provide Galaxy “on-demand” instances over heterogeneous cloud infrastructures
Source: Gigascience. 2020 Apr 6;9(4):giaa033. doi: 10.1093/gigascience/giaa033 (PMC7136032; doi:10.1093/gigascience/giaa033)
Supplement: giaa033_Reviewer_1_Report_Original_Submission — Kristian Peters -- 1/23/2020 Reviewed [file giaa033_reviewer_1_report_original_submission.pdf]

Reviewer Report

Title: Laniakea: an open solution to provide Galaxy â€œon-demandâ€� instances over heterogeneous cloud infrastructures.

Version: Original Submission **Date:** 1/23/2020

Reviewer name: Kristian Peters

Reviewer Comments to Author:

The current version has been greatly improved when compared to the last revision. The article has been cleared and is now well structured and easy to understand. All remarks of the reviewers have been resolved sufficiently enough. The presented cloud infrastructure (software) is now well documented and the proof of principle is working. I tested it out this morning and the deployment process and tearing down the deployed instances did work without any problems. One thing that I could not find in the article was whether Galaxy jobs are running within the deployed environment or in an agile way distributed throughout the cloud infrastructure. Other than minor English glitches I do not have any other remarks.

Level of Interest

Please indicate how interesting you found the manuscript: Choose an item.

Quality of Written English

Please indicate the quality of language in the manuscript: Choose an item.

Declaration of Competing Interests

Please complete a declaration of competing interests, considering the following questions:

- Have you in the past five years received reimbursements, fees, funding, or salary from an organisation that may in any way gain or lose financially from the publication of this manuscript, either now or in the future?
- Do you hold any stocks or shares in an organisation that may in any way gain or lose financially from the publication of this manuscript, either now or in the future?
- Do you hold or are you currently applying for any patents relating to the content of the manuscript?
- Have you received reimbursements, fees, funding, or salary from an organization that holds or has applied for patents relating to the content of the manuscript?
- Do you have any other financial competing interests?
- Do you have any non-financial competing interests in relation to this paper?

If you can answer no to all of the above, write 'I declare that I have no competing interests' below. If your reply is yes to any, please give details below.

I have no competing interests

I agree to the open peer review policy of the journal. I understand that my name will be included on my report to the authors and, if the manuscript is accepted for publication, my named report including any attachments I upload will be posted on the website along with the authors' responses. I agree for my report to be made available under an Open Access Creative Commons CC-BY license (http://creativecommons.org/licenses/by/4.0/). I understand that any comments which I do not wish to be included in my named report can be included as confidential comments to the editors, which will not be published.

Choose an item.

To further support our reviewers, we have joined with Publons, where you can gain additional credit to further highlight your hard work (see: https://publons.com/journal/530/gigascience). On publication of this paper, your review will be automatically added to Publons, you can then choose whether or not to claim your Publons credit. I understand this statement.

Yes Choose an item.
